# Alteration of Musashi1 Intra-cellular Distribution During Regeneration Following Gentamicin-Induced Hair Cell Loss in the Guinea Pig Crista Ampullaris

**DOI:** 10.3389/fncel.2019.00481

**Published:** 2019-10-25

**Authors:** Makoto Kinoshita, Chisato Fujimoto, Shinichi Iwasaki, Akinori Kashio, Yayoi S. Kikkawa, Kenji Kondo, Hideyuki Okano, Tatsuya Yamasoba

**Affiliations:** ^1^Department of Otolaryngology and Head and Neck Surgery, Faculty of Medicine, University of Tokyo, Tokyo, Japan; ^2^Department of Physiology, Keio University School of Medicine, Tokyo, Japan

**Keywords:** hair cell, supporting cell, inner ear, vestibule, regeneration, Musashi1

## Abstract

The mechanism underlying hair cell (HC) regeneration in the mammalian inner ear is still under debate. Understanding what molecules regulate the HC regeneration in mature mammals will be the key to the treatment of the inner ear disorder. Musashi1 (MSI1) is an RNA binding protein associated with asymmetric division and maintenance of stem cell function as a modulator of the Notch-1 signaling pathway. In this study, we investigated the cellular proliferative activity and changes in spatiotemporal pattern of MSI1 expression in the gentamicin (GM)-treated crista ampullaris (CA) in guinea pigs. Although the vestibular HCs in the CA almost disappeared at 14 days after injecting GM in the inner ear, the density of vestibular HCs spontaneously increased by up to 50% relative to controls at 56 days post-GM treatment (PT). The number of the type II HCs was significantly increased at 28 days PT relative to 14 days PT (*p* < 0.01) while that of type I HCs or supporting cells (SCs) did not change. The number of SCs did not change through the observational period. Administration of bromodeoxyuridine with the same GM treatment showed that the cell proliferation activity was high in SCs between 14 and 28 days PT. The changes in spatiotemporal patterns of MSI1 expression during spontaneous HC regeneration following GM treatment showed that MSI1-immunoreactivity was diffusely spread into the cytoplasm of the SCs during 7–21 days PT whereas the expression of MSI1 was confined to the nucleus of SCs in the other period. The MSI1/MYO7A double-positive cells were observed at 21 days PT. These results suggest that regeneration of vestibular HCs might originate in the asymmetric cell division and differentiation of SCs and that MSI1 might be involved in controlling the process of vestibular HC regeneration.

## Introduction

Damage and loss of sensory hair cells (HCs) in the inner ear through aging, exposure to noise, and genetic disorders cause hearing and balance disorders in millions of people each year (Nadol, 1993). While HCs can regenerate in both the auditory and vestibular systems in non-mammalian vertebrates (Cotanche, 1987; Cruz et al., 1987; Adler and Raphael, 1996; Adler et al., 1997), in mammals, spontaneous HC regeneration is very limited in the vestibular end organs (Forge et al., 1993; Warchol et al., 1993; Burns et al., 2012; Golub et al., 2012) and no spontaneous regeneration has been observed in the mature cochlea (Hawkins et al., 1976; Oesterle et al., 2008).

The mechanism underlying HC regeneration is a large topic of investigation, and generally two different mechanisms have been proposed as spontaneous HC regeneration models; mitotic cell division and non-mitosis-mediated direct transdifferentiation. In non-mammalian vertebrates, mitotic cell division occurs when a supporting cell (SC) divides and, subsequently, one or both daughter cells becomes a HC (Corwin and Cotanche, 1988; Ryals and Rubel, 1988; Jones and Corwin, 1996). Non-mitosis-mediated direct transdifferentiation refers to a cell fate change when SCs directly convert into HCs without cell division (Adler and Raphael, 1996; Baird et al., 1996; Jones and Corwin, 1996).

Recently, a study revealed that spontaneous HC regeneration occurred in the neonatal mouse cochlea after damage by genetic methods *in vivo*, and suggested that regenerated HCs were derived by both mitotic cell division and direct transdifferentiation (Bramhall et al., 2014; Cox et al., 2014). However, the mechanism underlying spontaneous regenerative process of HCs in the mature mammalian inner ear after damage is still controversial. Furthermore, when the mitotic cell division is involved in the mechanism of regenerative process, it is still unknown whether the type of cell division is symmetric or asymmetric. Symmetric cell division generates daughter cells with equivalent fates, whereas asymmetric cell division generates two daughter cells with different cell fates. Typically, in an asymmetric division, the stem or progenitor cell generates a copy of itself, which retains self-renewal ability and differentiation potential, and one daughter cell that enters the path of differentiation (Gómez-López et al., 2014).

Musashi 1 (MSI1), which is one of the highly conserved RNA-binding proteins, is expressed in embryonic neural progenitor cells and adult neural stem cells. MSI1 is a molecule required for asymmetric cell division of sensory organ precursor cells in *Drosophila* (Nakamura et al., 1994), and has been postulated to play important role in the maintenance and differentiation of stem cells (Okano et al., 2005). In mammals, MSI1 is considered to act as a Notch activator through translational repression of an intracellular Notch antagonist, m-Numb, and regulating cell differentiation during asymmetric cell division (Okano et al., 2002, 2005). During retinal cell development and retinal regeneration after asymmetric cell division *in vivo*, MSI1 immunoreactivity shifts from exclusively cytoplasmic in neural progenitor cells to predominantly nuclear localization in differentiating neurons (Kaneko and Chiba, 2009; Nickerson et al., 2011). These shifts in subcellular MSI1 localization characterize early binary fate for cell proliferation and differentiation. In the vestibular system, a previous study showed that gradual disappearance of MSI1 in HC but persistent presence of MSI1 in SC during development in mice (Sakaguchi et al., 2004). Therefore, we hypothesized that if the early regeneration process of vestibular HCs mainly depends on the asymmetric cell division, intracellular MSI1 expression pattern in progenitor cells would change during HC regeneration.

In the present study, we investigated the cellular proliferative activity after HC loss by gentamicin (GM) treatment in the mature vestibular end organs in guinea pigs to assess the occurrence of mitotic cell division in spontaneous regenerative process of HCs. Then, we assessed changes in the expression patterns of MSI1 in the HC regenerative process after HC loss to elucidate the type of cell division in the HC regenerative process.

## Materials and Methods

### Animals

Male Hartley guinea pigs, 4 weeks of age and weighing 250–300 g, were used in this study. All animals were bred and housed in the standard animal facility under normal guinea pig rearing conditions, and experiments were done according to guidelines of the University Committee for the Use and Care of Animals, University of Tokyo (approval number P11-034; Tokyo, Japan), and the National Institutes of Health Guide for the Care and Use of Laboratory Animals.

### Experimental Protocols

#### Experiment 1: Study to Examine the Time Course of HC Regeneration

We assessed the degree of vestibular HC damage 14 days after ototoxic GM treatment in different concentration: saline as control, 0.2 mg/ml, 0.4 mg/ml, 0.8 mg/ml, and 1.2 mg/ml. The animals received 0.2 ml GM administration through the cochleostomy in the left ear under the surgical microscope (M320 F12, Leica, Germany) after intramuscular injection of ketamine 40 mg/kg and xylazine 0.5 mg/kg. A concentration of 0.4 mg/ml was applied to this experiment as 0.4 mg/ml was damaging most HCs and there was no change at higher concentrations (Supplementary Figure S1).

The animals which received GM (0.4 mg/ml, 0.2 ml) treatment were euthanized at 7, 14, 21, 28, and 56 days (*n* = 6 each) post-GM treatment (PT). Six untreated animals were euthanized just before the start of experiment (0 day) and served as controls. Thereafter, the HCs and SCs in the harvested crista ampullaris (CA) were stained immunohistologically.

#### Experiment 2: Study to Examine Mitotic Activity During Spontaneous HC Regeneration

The animals (*n* = 18) which received the same GM administration as Experiment 1 were implanted micro-osmotic pumps (Model 2ML2, Alzet, Cupertino, CA, USA) subcutaneously in the interscapular region to deliver bromodeoxyuridine (BrdU, Sigma-Aldrich, Ireland) at 125 μg/h for 5 days (1–5 days, 6–10 days, and 11–15 days PT; *n* = 6 each). Each group was euthanized at 28 days PT. Unaffected side served as controls. Thereafter, the BrdU positive cells were confirmed immunohistologically.

#### Experiment 3: Study to Examine the Alteration of MSI1 Distribution During HC Regeneration

The animals (*n* = 30) which received the same GM treatment as Experiment 1 were euthanized at 7, 14, 21, 28, and 56 days (*n* = 6 each) PT. Six untreated animals served as controls. Thereafter, the change in the distribution of the Msi1 positive cells in the harvested CA were analyzed immunohistopathologically.

### Tissue Preparation

The collected temporal bones were fixed in 4% paraformaldehyde/phosphate-buffered saline (PBS; pH 7.4) for 2 h, and then harvested the CA under the microscope (SZX9, Olympus, Japan). The fixed specimens were immersed in PBS with 30% sucrose for 6 h, embedded in 5% agarose (type IX-A, Sigma-Aldrich, Ireland) and 20% sucrose in PBS, frozen in n-hexane (−60°C). These specimens were cut vertically into 15 μm thick sections from planum semilunatum to the center of the crista on a cryostat (Tissue-Tek Cryo3, Sakura Finetek, Japan; Kanda et al., 2008). At intervals of 45 μm, five sections including the center of CA were multiple-immunostained in each experiment and observed under a confocal microscope (A1^+^, Nikon, Japan; Supplementary Figure S2).

### Immunohistochemistry

Agarose embedded cryosections of CAs were washed in 0.1× PBS, and then placed in blocking solution (5% goat serum albumin and 0.1% Triton-X 100 in 0.1× PBS) for 30 min at room temperature. Sections were transferred into diluent (5% goat serum albumin and 0.1% Triton-X 100 in 0.1× PBS) containing primary antibodies for 1-h at 37°C. After PBS rinses, these sections were incubated in Alexa Fluor fluorescent secondary antibodies (1:100; Molecular Probes, USA) for 1-h at 37°C. After PBS rinses, these sections were mounted in Vectashield Mounting Medium with DAPI (Vector Laboratories Inc., Burlingame, CA, USA) and slides were coverslipped. These sections were observed under a confocal microscope. For BrdU staining, sections were placed in 2 N HCL for 20 min at room temperature before blocking process. The primary antibodies used were polyclonal rabbit anti-MYO7A (1:200; Proteus BioSciences, Ramona, CA, USA), monoclonal mouse anti-parvalbumin (Pvalb, 1:100; Sigma-Aldrich, Ireland), monoclonal rat anti-BrdU (1:200; AbD Serotec, UK), monoclonal rat anti-MSI1 (1:100; Medical & Biological Laboratories, Austin, TX, USA), and secondary antibodies were goat anti-rabbit Alexa Fluor 568, goat anti-mouse Alexa Fluor 488, goat anti-rat Alexa Fluor 568, goat anti-rabbit Alexa Fluor 488, goat anti-rat Alexa Fluor 488. For negative controls, an absorption test was performed. Ventricular zones of mouse embryonic brain (E14.5) known to express MSI1 served as positive controls.

For the Experiment 2, CAs were fixed in 0.1× PBS-buffered 4% paraformaldehyde for 24 h, and decalcified with 10% EDTA solution (pH 7.0). After embedding in paraffin, 4-μm sections were cut and mounted on silane-coated slides. Deparaffinized sections were autoclaved in antigen retrieval solution (Target Retrieval Solution, Dako, Tokyo, Japan) at 121°C for 20 min for antigen retrieval. The sections were treated with 3% hydrogen peroxide to block endogenous peroxidase activity and placed in 2 N HCL for 20 min at room temperature before the blocking process.

Immunohistochemistry was performed using monoclonal rat anti-BrdU (1:200; AbD Serotec, UK). Immunoreaction was detected using Histofine Simple Stain MAX-PO (Rat; Nichirei Corp., Tokyo, Japan). These sections were examined under light microscope with high-power fields (400×).

### Confocal Microscopy

A series of images were obtained using a Nikon A1^+^ confocal imaging system. For all the specimens, laser power settings and pinhole were not varied. Both amplifiers offset and detector gain were optimized for each end organ to prevent saturation of pixels in the images. To avoid cross-talk between the different probes, specimens were sequentially excited with the respective lasers and recorded. Minimal adjustments to photodetector gain were necessary between different specimens to prevent saturation of pixels in any specimen, but gain was held constant for all optimal image slices in a given tissue section.

**Figure 1:**
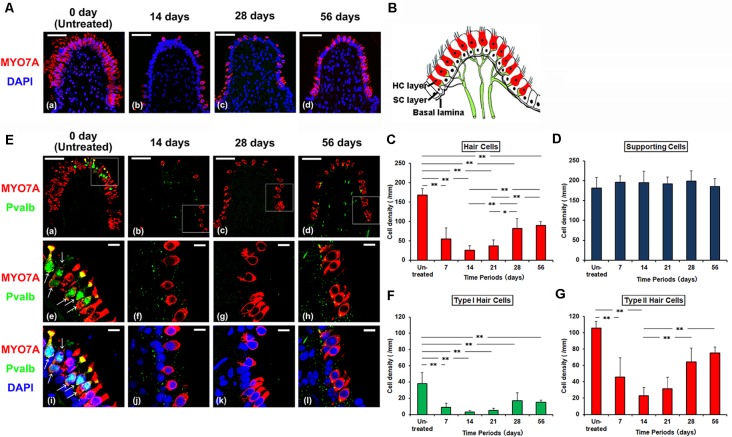
**(A)** Histologic changes of supporting cells (SCs) and hair cells (HCs) of the crista ampullaris from untreated control **(a)**, 14 days **(b)**, and 28 days **(c)** and 56 days **(d)** after gentamicin (GM) treated. Red represents anti-Myosin7a (MYO7A) labeling HCs and blue represents DAPI labeling of ds-DNA in the nuclei of cells. Scale bar = 50 μm. **(B)** The schema of crista ampullaris. **(C)** The densities (Mean ± SD) of HCs were reduced at 7 and 14 days post GM treatment (PT), followed by an increase at 28 and 56 days PT. **(D)** The cell densities (Mean ± SD) of SCs in all the observation periods did not change. **(E)** Histologic changes of type I HCs and type II HCs from untreated control **(a)**, 14 days **(b)**, 28 days **(c)** and 56 days **(d)** after GM treated. Each middle **(e–h)** and bottom **(i–l)** panel magnifies the boxed area in the respective top panel **(a–d)**. Red represents anti-MYO7A labeling HCs, green represents anti-Parvalbumin (Pvalb) labeling nerve calyces of type I HCs (arrow), and blue represents DAPI labeling of ds-DNA in the nuclei of cells. Scale bar = 50 μm **(a–d)**. Scale bar = 10 μm **(e–l)**. **(F)** The densities (Mean ± SD) of type I HCs significantly reduced from 7 days PT to 56 days PT as compared with untreated control. **(G)** The densities (Mean ± SD) of type II HCs were reduced at 7 and 14 days PT, followed by an increase at 28 and 56 days PT. **p < 0.01, *p < 0.05.

HCs were identified by anti-MYO7A labeling of their cytoplasm. Type I HCs were identified by the basal location of their DAPI-labeled nuclei within the sensory cell layer, their flask shape, and anti-Pvalb labeling of their associated calyx afferent endings. Type II HCs were identified by the more apical location of their DAPI-labeled nuclei within the sensory cell layer and lack of associated anti-Pvalb labeling. SCs were identified by lack of their anti-MYO7A and anti-Pvalb labeling, and DAPI-labeled nuclear location adjacent to the basal lamina (Figure 1B; Lysakowski and Goldberg, 1997; Zheng and Gao, 1997; Simmons et al., 2010).

### Statistical Analysis

Each cell density in Experiment 1 was calculated by the following method: on the digital photomicrographs, the number of each specific cell was counted in five sections per animal by unbiased investigators, and the length of basal lamina was measured by tracing with a calibrated computer mouse (Photoshop CS5 software, Adobe Systems, San Jose, CA, USA). Basal lamina was identified as a thin membrane under SCs. Each specific cell density was calculated by dividing the number of each specific immuno-positive cell by the length of basal lamina.

In Experiment 2, the target cell positive rates were calculated by dividing BrdU positive cells or BrdU/MYO7A double-positive cells by DAPI positive cells in the sensory epithelial layer on affected ear. In Experiment 3, the target cell positive rates were calculated by dividing MYO7A positive cells or MYO7A/MSI1 double-positive cells by DAPI positive cells in the sensory epithelial layer on affected ear.

SigmaPlot 11 statistical software (Systat Software Inc., San Jose, CA, USA) was used and all data were expressed as mean ± SD. Mean cell densities were compared between groups in each experiment. For these quantitative measures, statistical differences were examined using one-way analysis of variance (ANOVA). Differences with *p*-values of 0.05 or less after sequential Bonferroni adjustment for multiple tests were considered significant.

## Results

### Increased HC Density in the Vestibular Epithelium After GM-Induced Damage

After GM treatment, the density of MYO7A-positive HCs in CA markedly decreased until 14 days PT (Figures 1A,C). At 14 days PT, almost all of HCs were damaged. After 14 days PT, the density of HCs gradually increased.

Then, the types of increased vestibular HCs were analyzed. In untreated controls, type I HCs labeled with anti-MYO7A/Pvalb/DAPI and type II HCs labeled with anti-MYO7A/DAPI could both be identified by their more superficially located nuclei than those of SCs which formed a single row over the basal membrane. At 14 days PT, there was an almost complete disappearance of all HCs. Almost all of type I HCs disappeared at this time period (4% of the untreated period), and type II HCs were very few (20% of the untreated period; Figures 1E–G). At 56 days PT, type I HCs remained significantly fewer than at the untreated period (25% of the untreated period, *p* < 0.01). However, type II HCs increased in density at 56 days PT (82% of the untreated period) and there was no significant difference in density between at the untreated period and at 56 days PT. These cells formed a single layer with their nuclei located above the SCs (Figure 1E). These findings demonstrated that the vestibular HCs increased after GM-induced HC damage and the increased HCs were mainly type II HCs (Supplementary Table S1).

On the other hand, the density of SCs at the basal part of the sensory epithelia did not change after GM treatment (Figures 1A,D). In all the observation periods after GM treatment, the density of SCs showed no significant difference in comparison with the untreated period (Figures 1A,D, *p* > 0.05).

### Cellular Proliferative Activity in CA After GM Treatment

To investigate cellular proliferative activity in CA after GM treatment, BrdU were continuously injected at a low dose level by subcutaneous infusion at 1–5 days, 6–10 days, or 11–15 days PT, followed by evaluating the occurrence of BrdU-positive cells in the sensory epithelium by immunohistochemistry at 28 days PT.

**Figure 2:**
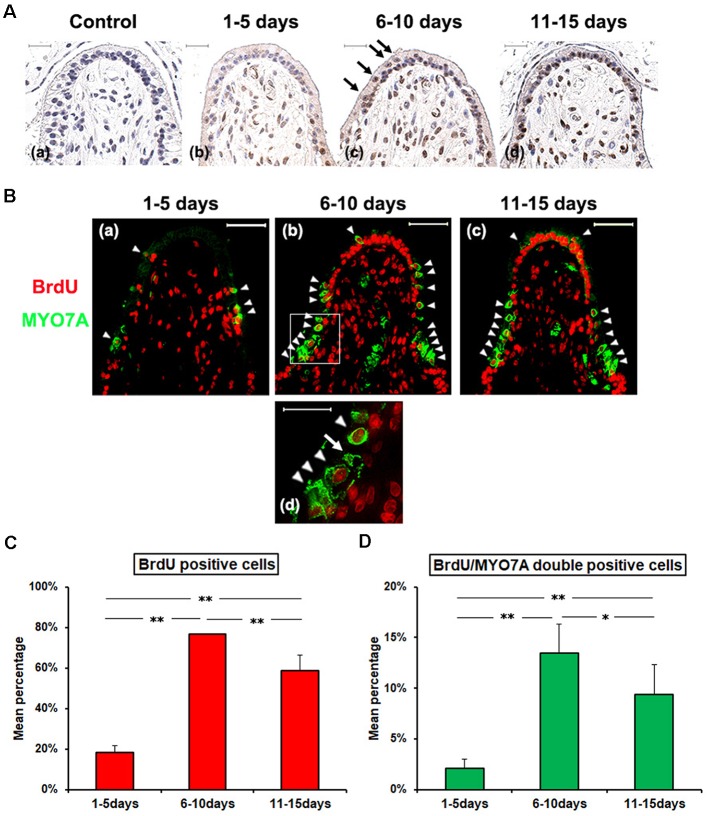
**(A)** Histopathologic evaluation of cellular proliferative activity with Bromodeoxyuridine (BrdU) infusion after GM treatment. Animals were divided into three groups by the BrdU infused period; 1–5 days **(b)**, 6–10 days **(c)**, and 11–15 days **(d)** PT. All animals were euthanized at 28 days PT. At 6–10 days PT and 11–15 days PT, many SCs with BrdU-positive nuclei were shown in the basal cell layer, and some BrdU positive cells were shown at the upper luminal portion of the epithelial layer **(c)**. **(B)** Fluorescence immunohistopathologic evaluation. Green represents anti-MYO7A labeling HC, and red represents anti-BrdU labeling, and white arrow heads point to a BrdU/MYO7A double-positive cells at the upper luminal portion of the epithelial layer. In specimens from the group dosed with BrdU on 1–5 days PT, there were few BrdU-positive cells detected in the sensory epithelium **(a)**. In the groups dosed on 6–10 days PT or on 11–15 days PT, a multitude of SCs with BrdU-positive nuclei were noted in the basal cell layer and also there were cells with BrdU taken up into their nuclei in the sensory cell layer **(b,c)**. The lower panel **(d)** magnifies the boxed area in the respective upper panel **(b)**. Scale bar = 50 μm. **(C)** The Mean percentage (Mean ± SD) of BrdU positive cells in the sensory epithelium. **(D)** The Mean percentage (Mean ± SD) of BrdU/MYO7A double-positive cells in the sensory epithelium. Both of BrdU positive cells and BrdU/MYO7A double-positive cells, there were significant differences among the three groups, with the highest value for the group dosed BrdU on 6–10 days PT. **p < 0.01, *p < 0.05.

The GM-untreated side did not show any BrdU-positive cells at 1–5 days PT, 6–10 PT or 11–15 PT (data not shown). As for the GM-treated side, there were few BrdU-positive cells detected in the sensory epithelium at 1–5 days PT (Figures 2A,B, Supplementary Table S2). At 6–10 days PT and 11–15 days PT, many SCs with BrdU-positive nuclei were shown in the basal cell layer of the vestibular epithelium, and some BrdU/MYO7A double-positive cells were shown at the upper luminal portion of the epithelial layer (Figure 2B). The percentage of BrdU-labeled nuclei was significantly higher at 6–10 days PT and 11–15 days PT than at 1–5 days PT (*p* < 0.01, Figure 2C). The percentage of BrdU/MYO7A double-positive cells was also significantly higher at 6–10 days PT and 11–15 days PT than at 1–5 days PT (*p* < 0.01, Figure 2D).

These results demonstrated that the activity of cell proliferation in CA was high at 6–15 days PT, and spontaneous HC regeneration after GM treatment occurred without association with the change in SC density.

### Changes in Intra-cellular Localization of MSIl During Spontaneous Regeneration Process of HCs

**Figure 3:**
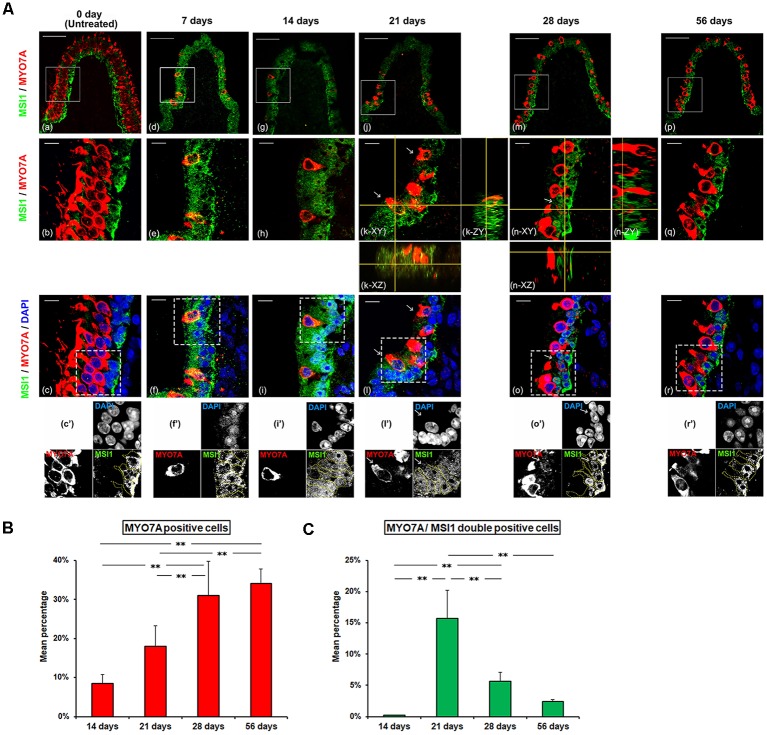
**(A)** Histological changes in intra-cellular localization of Musashi1 (MSIl) during spontaneous regeneration process of HCs. Each middle **(b,e,h,k,n,q)** and lower **(c,f,i,l,o,r)** panel magnifies the boxed area in the respective upper panel **(a,d,g,j,m,p)**. Each lowest panel **(c’,f’,i’,l’,o’,r’)** represents grayscale image of the dot-boxed area in the respective upper panel **(c,f,i,l,o,r)**. The area surrounded by the yellow dotted line represents SCs. Red represents anti-MYO7A labeling HCs, green represents anti-MSI1 labeling, and blue represents DAPI labeling of ds-DNA in the nuclei of cells. In untreated sections **(a–c)**, SCs showed positive MSI1-immunoreactivity confined to their nuclei while HCs were negative. At 7 and 14 days PT, HCs were no longer existent, and SCs were diffusely immunostained with MSI1 **(d–i)**. At 21 and 28 days PT **(j–o)**, MYO7A-positive HCs, with nuclei frequently positive for MSIl, were recognized (arrow). MSI1-immunoreactivities of SCs were gradually returned to the nuclear predominance. At 56 days PT **(p–r)**, MSI1 was nuclear predominance of SC. MYO7A-positive cells were frequently negative for MSI1. Scale bar = 50 μm **(a,d,g,j,m,p)**, Scale bar = 10 μm **(b,c,e,f,h,i,k,l,n,o,q,r)**. **(B)** The change of mean percentage (Mean ± SD) of MYO7A positive cells. MYO7A positive cells showed a progressive increase with significant differences throughout the period from 14 to 56 days PT. **(C)** The changes of mean percentage (Mean ± SD) of MSI1/MYO7A double-positive cells. MYO7A/MSI1 double-positive cells increased significantly during the period from 14 to 21 days PT, followed by a significant decrease from 21 to 56 days PT. **p < 0.01.

In the GM-untreated side, HCs were not labeled for MSIl while SC nuclei showed positive immunoreactivity for MSI1 (Figures 3Aa–c). As for the GM-treated side, MYO7A-positive cells were very few, and the nuclei and cytoplasm of SCs were diffusely immunostained for MSI1 at 7 days PT and 14 days PT (Figures 3Ad–i). At 21 days PT, MYO7A-positive HCs with their nuclei positive for MSIl were detected, and their nuclei were found at the basal part of the sensory epithelia, which is closed to SCs. In these MYO7A/MSI1 double-positive cells, the immunoreactivity for MYO7A was localized in the cytoplasm and MSIl was localized in the nucleus. In SCs, the nuclei and cytoplasm were diffusely immunostained for MSI1 at 21 days PT (Figures 3Aj–l). At 28 days PT as well as at 56 days PT, immunoreactivity for MSI1 in SCs was localized to the nucleus, and MYO7A/MSI1 double-positive cells decreased in percentage relative to 14 days PT (Figures 3Am–r). MYO7A-positive but MSI-negative cells increased in percentage and were located above SCs. The percentage of MYO7A-positive cells gradually increased over time between 14 and 56 days PT (Figure 3B). On the other hand, while the percentage of MYO7A/MSI1 double-positive cells significantly increased between 14 days and 21 days PT (*p* < 0.01), they decreased thereafter (Figure 3C, Supplementary Table S3).

Table 1 summarizes the changes in HC and SC densities and immunoreactivity patterns of MYO7A and MSI1 during observation periods. Increase in MYO7A-positive HC density but constant SC density in CA after GM treatment and changes in the distribution of MSI1 in HCs and SCs strongly suggest that the HC regeneration in CA after GM treatment is mainly caused by asymmetric cell division of SCs.

**Table 1:** The summary of changes in hair cell and supporting cell densities over time, and changes in MYO7A and MSI1 expression patterns.

		Untreated	14 days	21 days	28 days	56 days
Hair cell	Cell density (%)	100	16	22	49	54
	MYO7A	C	C	C	C	C
	MSI1	-	-	N++	N+	-
Supporting cell	Cell density (%)	100	107	105	109	102
	MYO7A	-	-	-	-	-
	MSI1	N	N, C	N, C	N > C	N

*C, cytoplasm; N, nucleus*.

## Discussion

In the present study, we have examined the cellular proliferative activity after GM treatment in guinea pig and shown that the density of vestibular HCs increased spontaneously up to 50% of control. The increased vestibular HCs were mainly type II’s while the density of SCs did not change. A BrdU assay showed that the cell proliferation activity was high in SCs at 6–15 days PT. The distribution of MSI1-immunoreactivity changed from the nuclei to the cytoplasm in SCs between 14 days and 28 days PT. These results suggest that the regeneration process of HCs in CA mainly involves asymmetric cell division of SCs and the differentiation into HCs.

Immunohistochemical analyses in this study indicated that the majority of regenerated vestibular HCs were Type II. This result is consistent with previous studies (Forge et al., 1993, 1998; Golub et al., 2012). Forge et al. (1998) examined vestibular HCs in the mouse utricle after GM treatment and found that all new HCs possess short, thin stereocilia and basolateral presences and they lack calyceal afferent endings, indicating that only Type II HC are replaced even after long recovery periods. It is unclear why Type I HCs are not regenerated in mammals. In birds, for comparison, the full complement of Type I and Type II HCS is regenerated after ototoxic insult (Weisleder et al., 1995). Interestingly, the majority of regenerated vestibular HCs by Atoh1 gene transfer after neomycin treatment is Type I HCs (Xu et al., 2012), suggesting that the different mechanisms may regulate the regenerating process between Type I and Type II vestibular HCs.

Two different mechanisms have been hypothesized as models of spontaneous HC regeneration: non-mitosis-mediated direct transdifferentiation and mitotic cell division. Non-mitosis-mediated direct transdifferentiation refers to a cell fate change when SCs directly convert into HCs without cell division (Adler and Raphael, 1996; Baird et al., 1996; Jones and Corwin, 1996).

Several previous studies have shown that transdifferentiation contributes to HC regeneration. Roberson et al. (2004) examined the processes underlying the regeneration of HCs after GM treatment in avian auditory epithelium and observed cells with an intermediate morphology between the HC and SC phenotype. These cells were observed during the early period of regeneration and identified as resulting from direct transdifferentiation as there was no BrdU labeling (Roberson et al., 2004). Golub et al. (2012) examined proliferative activity of vestibular HCs after administration of diphtheria toxin (DT) in adult *Pou4f3^+/DTR^* mice, in which vestibular HCs can be specifically killed by injection of DT *in vivo*. They reported that new HCs did not incorporate BrdU, the number of SCs decreased, and SCs showed significant upregulation of the pro-HC gene *Atoh1*. Their results suggest that direct transdifferentiation from SCs into HCs is the main source of HC regeneration after DT-induced HC loss in adult mice.

On the other hand, mitotic cell division occurs when a SC divides and, subsequently, one or both daughter cells become HC (Corwin and Cotanche, 1988; Ryals and Rubel, 1988; Jones and Corwin, 1996). In mitotic cell division, new HCs can originate from HCs, SCs or uncommitted stem cells (Murata et al., 2004). Previous studies indicated that SCs are the progenitor cells for mitotic HC regeneration following damage (Forge et al., 1993; Warchol et al., 1993; Tsue et al., 1994; Rubel et al., 1995; Tanyeri et al., 1995; Yamane et al., 1995; Lang et al., 1996; López et al., 1998; Chen and Streit, 2013).

In the present study, MSIl expression was confined to the nuclei of SCs in the GM-untreated controls, suggesting that the SCs possess the functions like as an undifferentiated neural stem cell. MSIl located in the nucleus is thought to maintain adult-stage cells in their undifferentiation state (Sakakibara et al., 1996, 2002; Okano et al., 2005). After GM was administered, the cell proliferation activity evaluated by BrdU assay was high in SCs at 6–15 days PT. MSI1 was diffusely spread in the cytoplasm as wells in the nuclei in SCs at 14 days PT, when HC loss was most evident. A previous study revealed that BrdU-positive nuclei were observed in the mouse utricle after GM treatment (Kawamoto et al., 2009). Another previous study reported that diffuse spread of expression of MSI1 was observed during enhanced tumor cell proliferation in human glioblastoma and medulloblastoma cell lines (Muto et al., 2012). Our results suggest that SCs have a prosperous proliferative activity during this period. During 21–28 days PT, the MSI1 in SCs was gradually redistributed from the nucleus to the cytoplasm, and MSI1 was also observed in the nuclei of the MYO7A-labeled HCs in this period. At 56 days PT, MSI was still present in the nuclei of the SCs but there was no MSI1-immunoreactivity in the HCs. MSI1 protein plays an important role in asymmetric mitotic divisions by way of strong expression in neural stem cells and neural precursor cells (Imai et al., 2001). It has been demonstrated that MSI1 was intensely expressed in self-regenerating stem cells during the asymmetric division process of undifferentiated stem cells of the central nervous system, whereas in the differentiating cell lineage, MSI1 expression was extremely low and vanishes almost completely in a progressing cell differentiation process to nerve cells or glia cells (Sakakibara et al., 1996). In all these types of stem cells, MSI1 is redistributed from the cytoplasm to the nuclear compartment. Collectively, our results suggest that asymmetric cell divisions and differentiation of SCs might have a strong contribution to the process of mammalian vestibular HC regeneration and that MSIl might be involved in the cellular fate determination in vestibular SCs. Spontaneous HC regeneration without association with the change in SC density supported asymmetric cell division of SCs.

**Figure 4:**
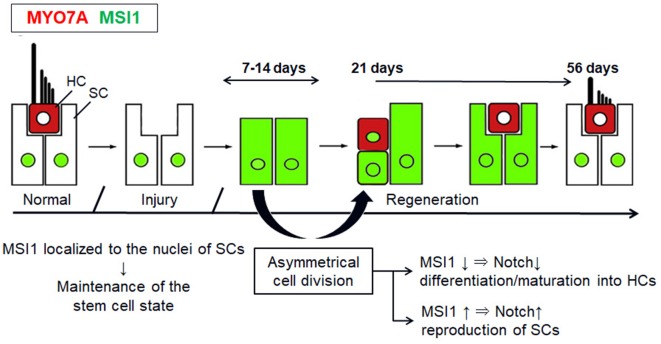
Schematic diagrams show the proposed process of changes of MSI1 and MYO7A in the regenerating vestibular epithelium. This is a modified figure from Wang et al. (2010).

Figure 4 shows a schematic diagram of the proposed process of spontaneous regeneration of vestibular HCs concomitant with the changes in the distribution of MSI1 and MYO7A based on this study. Our results suggest that asymmetric cell divisions and differentiation of SCs at least have some contribution to the process of mammalian vestibular HC regeneration and that MSIl might be involved in the cellular fate determination in vestibular SCs. The control of the endogenous neural stem cell differentiation and induction mechanism may become one of the treatment strategies for damage and loss for vestibular HCs in mammals.

In conclusion, we have examined the proliferative activity of vestibular HCs after GM treatment in guinea pig and have shown that the density of vestibular HCs increased spontaneously up to 50% of control. BrdU assay showed that the cell proliferative activity was high in SCs while the density of SCs did not change throughout the observational period. Changes in the spatiotemporal patterns of MSI1 expression during spontaneous HC regeneration following GM treatment showed that MSI1-immunoreactivity was diffusely spread in the cytoplasm of the SCs during 7–21 days PT whereas the expression of MSI1 was confined to the nucleus of SCs in the other period. The MSI1/MYO7A double-positive cells were observed at 21 days PT. These results suggest that regeneration of vestibular HCs might originate in the asymmetric cell division and differentiation of SCs and that MSI1 might be involved in controlling the process of vestibular HC regeneration.

## Data Availability Statement

All datasets generated for this study are included in the article/Supplementary Material.

## Ethics Statement

This study was carried out in accordance with the recommendations of the University Committee for the Use and Care of Animals, University of Tokyo, and the National Institutes of Health Guide for the Care and Use of Laboratory Animals. The protocol was approved by the University of Tokyo (approval number P11-034; Tokyo, Japan).

## Author Contributions

MK conceived the study, conducted the experiments, wrote the manuscript, and edited the manuscript for content. AK, YK, and KK provided technical advice and conducted the experiments. CF and SI conducted the experiments and edited the manuscript for content. HO and TY conceived the study and edited the manuscript for content.

## Conflict of Interest

The authors declare that the research was conducted in the absence of any commercial or financial relationships that could be construed as a potential conflict of interest.

## Supplementary Material

FIGURE S1 Hair cell densities in crista ampullaris 14 days after gentamicin treatment in different concentration.

FIGURE S2 Observed five sections of crista ampullaris.

TABLE S1 Alteration of each cell number after gentamicin treatment.

TABLE S2 Cellular proliferative activity in crista ampullaris after gentamicin treatment.

TABLE S3 Alteration in number of MYO7A and MYO7A/MSI1 positive cells during hair cell regeneration.
