# Supplementary figures and images for: Alteration of Musashi1 Intra-cellular Distribution During Regeneration Following Gentamicin-Induced Hair Cell Loss in the Guinea Pig Crista Ampullaris
Source: Front Cell Neurosci. 2019 Oct 25;13:481. doi: 10.3389/fncel.2019.00481 (PMC6824208; doi:10.3389/fncel.2019.00481)

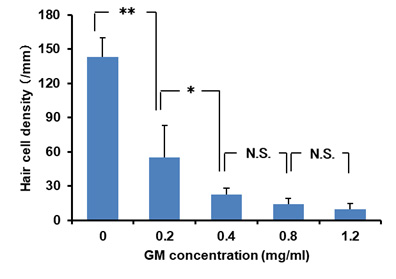

Supplement: TABLE S2 — Cellular proliferative activity in crista ampullaris after gentamicin treatment. [file Image_1.JPEG]

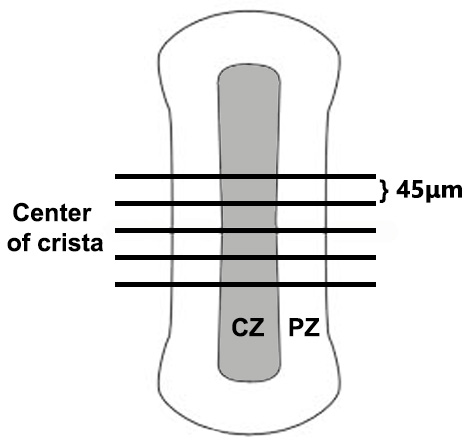

Supplement: TABLE S3 — Alteration in number of MYO7A and MYO7A/MSI1 positive cells during hair cell regeneration. [file Image_2.JPEG]
